# Propofol Alleviates Apoptosis Induced by Chronic High Glucose Exposure via Regulation of HIF-1*α* in H9c2 Cells

**DOI:** 10.1155/2019/4824035

**Published:** 2019-04-10

**Authors:** Jinjun Pu, Shun Zhu, Dandan Zhou, Lidong Zhao, Ming Yin, Zejian Wang, Jiang Hong

**Affiliations:** ^1^Department of Internal and Emergency Medicine, Shanghai General Hospital (Originally Named Shanghai First People's Hospital), Shanghai Jiao Tong University School of Medicine, Shanghai, China; ^2^Department of Emergency Medicine, Putuo Hospital Affiliated to Shanghai University of Traditional Chinese Medicine, Shanghai, China; ^3^School of Pharmacy, Shanghai Jiao Tong University, Shanghai, China

## Abstract

**Background:**

The sedative anesthetic, propofol, is a cardioprotective agent for hyperglycemia-induced myocardial hypertrophy and dysfunction in rats. However, the specific protective mechanism has not been clarified.

**Methods and Results:**

In this experiment, we used H9c2 cells subjected to 22 mM glucose lasting for 72 hours as an in vitro model of cardiomyocyte injury by hyperglycemia and investigated the potential mechanism of propofol against hyperglycemic stress in cells. Propofol (5, 10, or 20 *μ*M) was added to the cell cultures before and during the high glucose culture phases. Cell viability and levels of ROS were measured. The levels of proinflammatory cytokines were tested by ELISA. The levels of SIRT3, SOD2, PHD2, HIF-1*α*, Bcl-2, P53, and cleaved caspase-3 proteins were detected by western blotting. Our data showed that propofol attenuated high glucose-induced cell apoptosis accompanied by a decrease in the level of reactive oxygen species (ROS) and proinflammatory cytokines. Meanwhile, propofol decreased the apoptosis of H9c2 cells via increasing the expression of Bcl-2, SIRT3, SOD2, and PHD2 proteins and decreasing the expression of cleaved caspase-3, P53, and HIF-1*α*. Real-time PCR analysis showed that propofol did not significantly change the HIF-1*α* but increase PHD2 at mRNA level. HIF-1*α* silence significantly decreased apoptosis and inflammation in H9c2 cell during high glucose stress. Pretreatment of IOX2 (the inhibitor of PHD2) inhibited cell viability until the concentration reached 200 *μ*M during high glucose stress. However, 50 *μ*M TYP (the inhibitor of SIRT3) significantly inhibited cell viability during high glucose stress. Delayed IOX2 treatment for 6 hours significantly inhibited cell viability during high glucose stress.

**Conclusions:**

Propofol might alleviate cell apoptosis via SIRT3-HIF-1*α* axis during high glucose stress.

## 1. Introduction

Diabetes is one of the most common diseases affecting human health and bringing a large economic burden in modern society [1]. Studies have revealed that nearly 80% of diabetes-associated death were caused by cardiovascular diseases [2]. Previous studies have demonstrated that chronic hyperglycemia frequently causes cardiomyocyte dysfunction or apoptosis, eventually results in the development of heart failure [3]. Hyperglycemia is the metabolic hallmark of diabetes, which has been shown to promote excessive production of reactive oxygen species (ROS) [4, 5] and proinflammatory cytokines [6]. The ROS and inflammatory cytokines induce impairment in cardiac contractile function, promote myocardial apoptosis, and eventually induce the development of cardiac hypertrophy and heart failure [7, 8]. Therefore, therapeutic strategies aimed at reducing ROS levels through the inhibition of ROS production or increase of ROS scavenging may provide a promising method for the treatment of diabetic cardiovascular disease.

Propofol, one of the widely used intravenous anesthetics, has been shown to possess pleiotropic effects such as antioxidant, anti-inflammatory, and cardioprotective function [9, 10]. It has been shown that propofol reduces oxidative stress and inhibits the release of proinflammatory cytokines such as IL-6 and TNF-*α* in both *in vitro* and *in vivo* settings [11, 12]. In addition, propofol has also been shown to attenuate high glucose-induced hypertrophy and apoptosis in cardiomyocytes and reduce levels of ROS and malondialdehyde production [13]. Although the cardioprotective effects of propofol have been clearly defined by our group and others, the mechanism remains poorly described.

Sirtuins belong to a conserved family of NAD-dependent ADP ribosyltransferases and protein deacetylases and has been reported to be involved in many biological activities and processes including metabolism, stress responses, and longevity [14]. Sirtuin-3 (SIRT3), a mitochondria NAD^+^-dependent deacetylase, is reported to destabilize HIF-1*α* via PHD2 [15] and protect endothelial cells damage induced by high glucose exposure [16]. To date, the connection between propofol and SIRT3 and its downstream signaling pathways during high glucose stress has not yet been established. Therefore, we hypothesize that the cardioprotective effect of propofol is at least partially attributed to its antioxidant properties via the regulation of the HIF-1*α* signal pathway. In this study, we chose a high glucose medium-cultured H9c2 cell line as a model of hyperglycemia-induced cardiomyocyte injury and investigated the potential mechanism of propofol against hyperglycemic stress in cells and evaluated the effect of propofol on high glucose-induced apoptosis as well as cellular ROS level and proinflammatory cytokines by investigating the SIRT3/PHD2/HIF-1*α* signal pathway systemically.

## 2. Materials and Methods

### 2.1. Cell Culture

The H9c2 cells, a cardiomyoblast cell line originally derived from the rat left ventricle, were purchased from Shanghai Institute for Biological Sciences, Chinese Academy of Sciences (Shanghai, China). The cells were cultured in low glucose (5.5 mM) minimum essential medium (Gibco-Invitrogen, Grand Island, NY, USA) supplemented with 10% fetal bovine serum (Gibco-Invitrogen, Grand Island, NY, USA). Cells were maintained in a humidified atmosphere consisting of 5% CO_2_ and 95% air at 37°C. The medium was updated every 2 days. To establish high glucose- (HG-) induced stress model in H9c2 cells, D-glucose (Sinopharm Chemical Reagent Co. Ltd., Shanghai, China) was added in culture medium to reach the final concentration of 22 mM glucose. The concentration of 5.5 mM glucose was used as the control group. A dose-dependent effect of propofol was evaluated by adding 5, 10, 20, and 40 *μ*M of propofol to the cells, respectively [17]. IOX2 was purchased from Selleckchem and dissolved in DMSO at the concentration of 100 mM as a stock solution. The final concentration of DMSO in culture medium is less than 0.5%. To evaluate the effect of IOX2 on the protective of propofol, H9c2 cells were seeded in 96-well cell culture plates 6 hours before or after propofol treatment.

### 2.2. Cell Viability

Cell viability was determined by 3-(4,5-dimethylthiazol-2-yl)-2,5-diphenyltetrazolium bromide (MTT) assay (Beyotime, Haimen, China). Briefly, 10 *μ*L of MTT solution was added to cells to reach a final concentration of 0.5 mg/mL and incubated for 4 h at 37°C. After removing the culture medium, 100 *μ*L of DMSO was added to dissolve formazan crystals and the absorbance was read at 570 nm using an AMR-100 automatic enzyme analyzer (Allsheng, Hangzhou, China).

### 2.3. Detection of Intracellular ROS

Intracellular ROS levels were assessed using 2′,7′-dichlorofluorescin diacetate (DCFH-DA, Beyotime, Haimen, China), which forms dichlorofluorescein, fluorescent compound with ROS. Cells were preloaded with 10 *μ*M DCFH-DA for 20 min at 37°C, and then the plates were washed using MEM without serum five times at least. Fluorescence was determined using microplate reader with excitation/emission wavelength at 488/525 nm.

### 2.4. IL-1*β*, IL-6, and TNF-*α* Measurement Using ELISA

IL-1*β*, IL-6, and TNF-*α* production and secretion were determined in by ELISA in cell culture supernatant following the manufacturer's instructions (Beyotime Biotechnology, Shanghai, China). The results were from at least three experiments.

### 2.5. Apoptosis Assessment Using Flow Cytometry

To explore the rate of apoptosis in H9c2 cells during high glucose stress, an Apoptosis Detection Kit (Beyotime Biotechnology, Shanghai, China) was used following the procedures. Briefly, cells were trypsinized and resuspended at a concentration of 1 × 10^6^/mL in diluted binding buffer and labeled with 10 *μ*L of annexin V-FITC. Cells were incubated 30 min at room temperature in the dark, and 5 *μ*L of PI was added for 5 min followed by adding 400 *μ*L of 1× binding buffer into each tube. Flow cytometric analysis was performed to monitor the annexin Vin green and the DNA bound PI in red. Data acquisition and analysis were performed using the FlowJo software.

### 2.6. Western Blotting

Protein were extracted using RIPA buffer (50 mM Tris, pH 7.4, 150 mM NaCl, 1% Triton X-100, 1% sodium deoxycholate, 0.1% SDS) containing protease and phosphatase inhibitor cocktails (Roche, Germany). The supernatants were collected after centrifugation, and protein concentration was determined with bicinchoninic acid (BCA) kit (Beyotime, Haimen, China) according to the manufacturer's instructions. Then 30 *μ*g of total protein was loaded into 10% SDS-PAGE gel and transferred to polyvinylidene difluoride membranes (PVDF, Millipore, Billerica, MA, USA), and 5% of the BSA in TBS containing 0.1% Tween-20 (TBS-T) was used to block the membrane at room temperature for 1 hour. Primary antibodies were added and incubated at 4°C for overnight. Bcl-2, p53, and cleaved caspase-3 antibodies were purchased from Cell Signaling (Danvers, MA, USA); SIRT3, SOD2, PHD2, and HIF-1*α* were obtained from Abcam (Cambridge, UK); GAPDH was from Protein Tech Group (Chicago, IL, USA) and used as an internal control. After primary antibody, the membranes were washed in TBS-T for three times and horse radish peroxidase-conjugated secondary antibodies (Protein Tech Group, Chicago, IL, USA) were added to the membranes at a dilution of 1: 5000 for 1 hour at room temperature. The density of immunoblotting bands was shown via enhanced chemiluminescent (ECL) substrate (Thermo Pierce, Rockford, IL, USA).

### 2.7. Quantitative Real-Time PCR

Total RNA from the cells was extracted using TRIzol reagent (Wuhan Servicebio Technology Co. Ltd., Wuhan, China), and first-strand cDNA was synthesized using a reverse transcription (RT) kit (Thermo, USA). Quantitative real-time PCR was carried out on Step One Plus Real-Time PCR System (Applied Biosystems, Foster City, CA, USA) using SYBR Green master mix (FastStart Universal SYBR Green Master, ABI, USA). The sequence of the primers is listed in Table 1. The relative gene expression levels were analyzed using the 2^−ΔΔCt^ method, and all genes were normalized to GAPDH.

### 2.8. Short Interfering RNA (siRNA) Transfection for Hypoxia-Inducible Factor-1*α* (HIF-1*α*) Silencing

H9c2 cells were seeded in 6-well plates at a density of 5 × 10^6^ cells/well 16 hours before transfection. Three pairs of siRNA (50 nM) against HIF-1*α* were transfected into H9c2 cells with Lipofectamine 2000 to knockdown HIF-1*α*. The scrambled siRNA was used as control (provided by the Shanghai Tuo Ran Biological Company). Six hours of posttransfection, fresh growth medium was added to the cells for additional 3 days with or without the treatment of propofol. Cells were then harvested for detection of the mRNA and protein levels of HIF-1*α* by real-time PCR and western blotting. All the sequence of siRNAs is listed in Table 2.

### 2.9. Statistical Analysis

Data collected from 3~6 independent experiments were used to calculate the mean value. The results were presented as the mean ± standard error of mean (SEM). Statistical analysis was performed using Statistical Package for the Social Sciences (SPSS, Chicago, IL, USA). Quantitative data were analyzed by one-way analysis of variance (ANOVA). The Student-Newman-Keuls test was used for post hoc analysis to identify significant differences between groups. *P* < 0.05 was considered as statistically significant.

## 3. Results

### 3.1. The Effect of Propofol on the Cell Viability during High Glucose Stress

**Figure 1:**
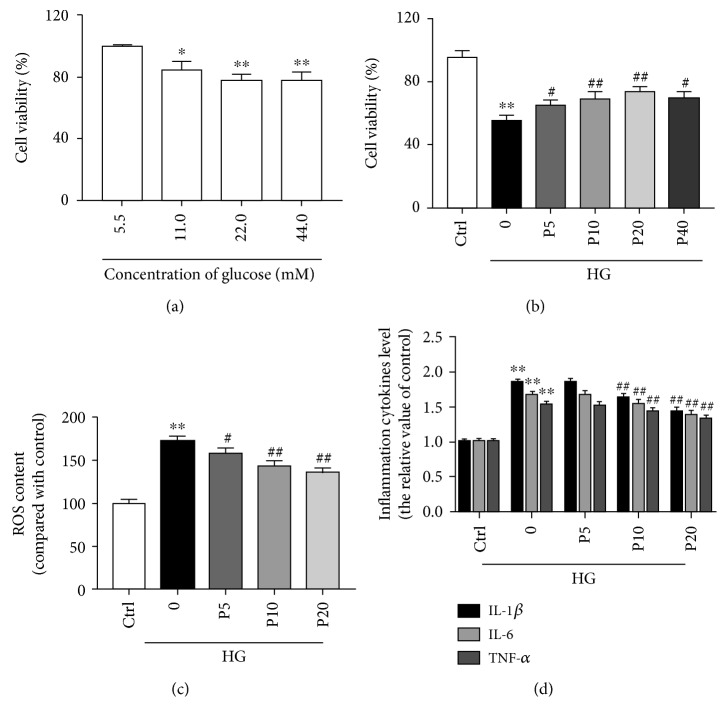
Protective effects of propofol against high glucose stress in H9c2 cells. Propofol (5, 10, 20, and 40 *μ*M) adopted during the entire high glucose culture phase. The control group was defined as 100%. (a) Effect of different concentrations of glucose on cell viability in H9c2 cells. Cell viability was assessed by measuring the MTT reduction. (b) Effects of different concentrations of propofol on cell viability under high glucose conditions in H9c2 cells. (c) Effects of propofol on high glucose-induced intracellular ROS levels in H9c2 cells. (d) Effects of different concentrations of propofol on the secretion of proinflammatory cytokines induced by high glucose in H9c2 cells. The results were shown as mean ± SEM from five independent experiments. ^∗^*P* < 0.05, ^∗∗^*P* < 0.01, and ^∗∗∗^*P* < 0.001 versus control; ^#^*P* < 0.05, ^##^*P* < 0.01, and ^###^*P* < 0.001 versus the high glucose-treated group without drugs. Ctrl = blank control group; 0 = high glucose model group; P5 = 5 *μ*M propofol pretreatment group; P10 = 10 *μ*M propofol pretreatment group; P20 = 20 *μ*M propofol pretreatment group; P40 = 40 *μ*M propofol pretreatment group; and HG = 22 mM high glucose culture.

Our data showed that with the addition of 11~44 mM glucose to the medium for 72 h, the cell vitality declined in a dose-dependent manner compared with normal glucose group. Twenty-two mM glucose causes obvious significant decrease in cell viability and 44 mM glucose did not further decrease it (Figure 1(a)). Therefore, we chose the concentration of 22 mM glucose for the following experiments to induce high glucose stress. The effect of propofol on the viability of H9c2 cells was measured by MTT assay. Compared to the high glucose only group, propofol increased cell viability in a dose-dependent manner at the range of 5~20 *μ*M. However, 40 *μ*M of propofol did not further improve cell viability during high glucose insult (Figure 1(b)).

### 3.2. Propofol Decreases ROS Levels Induced by High Glucose in H9c2 Cells

High glucose is known to induce oxidative stress. Indeed, we confirmed that high glucose obviously elevated intracellular ROS levels compared to the control group, whereas high glucose-induced ROS level elevation was obviously significantly inhibited with by the increasing doses of propofol (Figure 1(c)). This is consistent with previous findings on how propofol decreases ROS levels induced by OGD/R in H9c2 cells [17].

### 3.3. Propofol Decreases High Glucose-Induced Proinflammatory Factor Secretion in H9c2 Cells

As shown in Figure 1(d), after H9c2 cells were cultured with high glucose for 72 h, the levels of IL-6, IL-1*β*, and TNF-*α* were significantly increased in the media compared to the control group (*p* < 0.01), whereas, pretreatment of propofol at concentrations of 10 and 20 *μ*M significantly reduced these proinflammatory cytokine production (*p* < 0.01). Compared to the 10 *μ*M propofol group, the 20 *μ*M propofol group further inhibited inflammatory cytokines. These findings suggested that propofol protects the H9c2 cells against high glucose-induced cytotoxicity, through, at least in part, the inhibition of inflammatory response.

### 3.4. The Effect of TYP and IOX2 on the Cell Viability during High Glucose Stress

**Figure 2:**
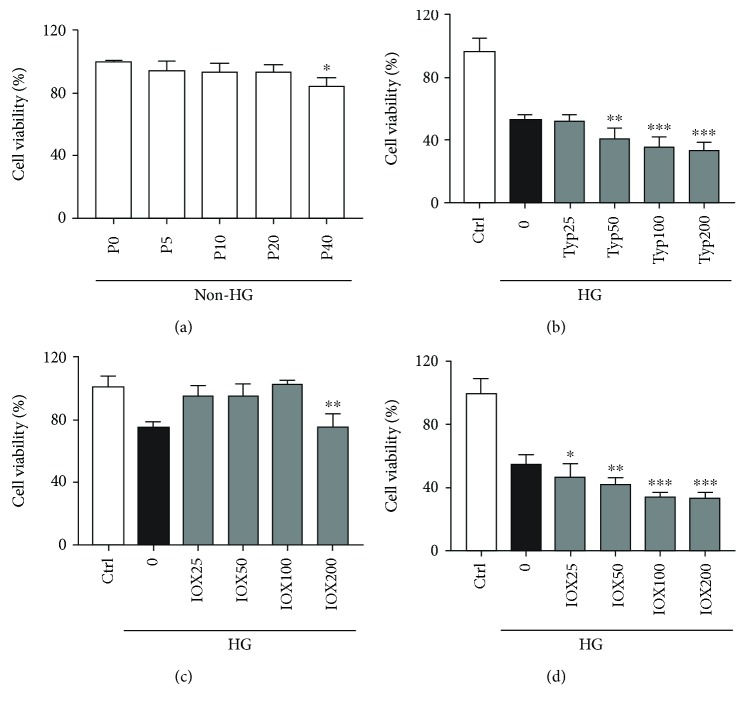
The effect of TYP and IOX2 on the cell viability during high glucose stress. (a) The effect of propofol on the cell viability under non-HG conditions. Propofol (5, 10, 20, and 40 *μ*M) adopted during the entire no high glucose culture phase. P0 = 0 *μ*M propofol pretreatment group; P5 = 5 *μ*M propofol pretreatment group; P10 = 10 *μ*M propofol pretreatment group; P20 = 20 *μ*M propofol pretreatment group; P40 = 40 *μ*M propofol pretreatment group; and non-HG = 5.5 mM glucose culture. (b) Effects of different concentrations of TYP (the inhibitor of SIRT3) on cell viability under high glucose conditions in H9c2 cells. Ctrl = blank control group; 0 = high glucose model group; TYP25 = 25 *μ*M TYP pretreatment group; TYP50 = 50 *μ*M TYP pretreatment group; TYP100 = 100 *μ*M TYP pretreatment group; TYP200 = 200 *μ*M TYP pretreatment group; and HG = 22 mM high glucose culture. (c) Effects of different concentrations of IOX2 (the inhibitor of PHD2) on cell viability under high glucose conditions in H9c2 cells. Ctrl = blank control group; 0 = high glucose model group; IOX25 = 25 *μ*M IOX2 pretreatment group; IOX50 = 50 *μ*M IOX2 pretreatment group; IOX100 = 100 *μ*M IOX2 pretreatment group; IOX200 = 200 *μ*M IOX2 pretreatment group; and HG = 22 mM high glucose culture. (d) Effects of different concentrations of IOX2 (delayed 6-hour treatment) on cell viability under high glucose conditions in H9c2 cells. Ctrl = blank control group; 0 = high glucose model group; IOX25 = 25 *μ*M IOX2 treatment group; IOX50 = 50 *μ*M IOX2 treatment group; IOX100 = 100 *μ*M IOX2 treatment group; IOX200 = 200 *μ*M IOX2 treatment group; and HG = 22 mM high glucose culture. The results were shown as mean ± SEM from five independent experiments. ^∗^*P* < 0.05, ^∗∗^*P* < 0.01, and ^∗∗∗^*P* < 0.001 versus control; ^#^*P* < 0.05, ^##^*P* < 0.01, and ^###^*P* < 0.001 versus the high glucose-treated group without drugs.

Our data showed that with the pretreatment of 0~40 *μ*M propofol to the medium for 72 h under non-HG conditions, propofol did not change cell viability at the range of 5~20 *μ*M. However, 40 *μ*M of propofol decreased cell viability slightly (Figure 2(a)), and 50 *μ*M TYP pretreatment significantly inhibited cell viability during high glucose stress (Figure 2(b)). Meanwhile, IOX2 pretreatment inhibited cell viability until the concentration reaches 200 *μ*M during high glucose stress (Figure 2(c)). If IOX2 treatment was delayed for 6 hours, cell viability decreased in a dose-dependent manner during high glucose stress (Figure 2(d)).

### 3.5. Propofol Inhibited Cell Apoptosis Induced by High Glucose Stress in H9c2 Cells

**Figure 3:**
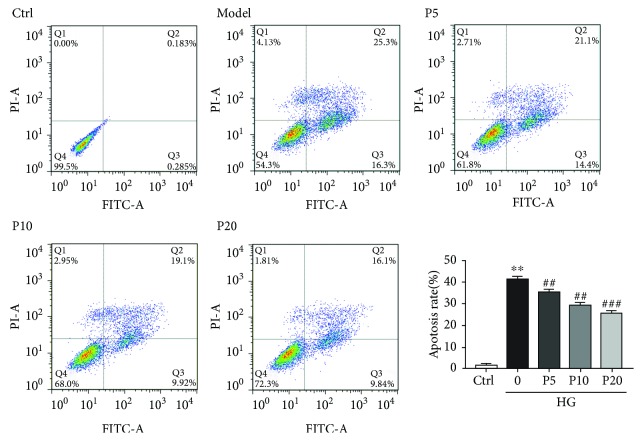
Propofol inhibited cell apoptosis induced by high glucose in H9c2 cells. Quantification of the apoptotic cell population by flow cytometry. Propofol decreased the percentage of apoptotic cells compared with the model. The results were shown as mean ± SEM from five independent experiments. ^∗^*P* < 0.05, ^∗∗^*P* < 0.01, and ^∗∗∗^*P* < 0.001 versus control; ^#^*P* < 0.05, ^##^*P* < 0.01, and ^###^*P* < 0.001 versus the high glucose-treated group without drugs. Ctrl = blank control group; 0 = high glucose model group; P5 = 5 *μ*M propofol pretreatment group; P10 = 10 *μ*M propofol pretreatment group; P20 = 20 *μ*M propofol pretreatment group; and HG = 22 mM high glucose culture.

**Figure 4:**
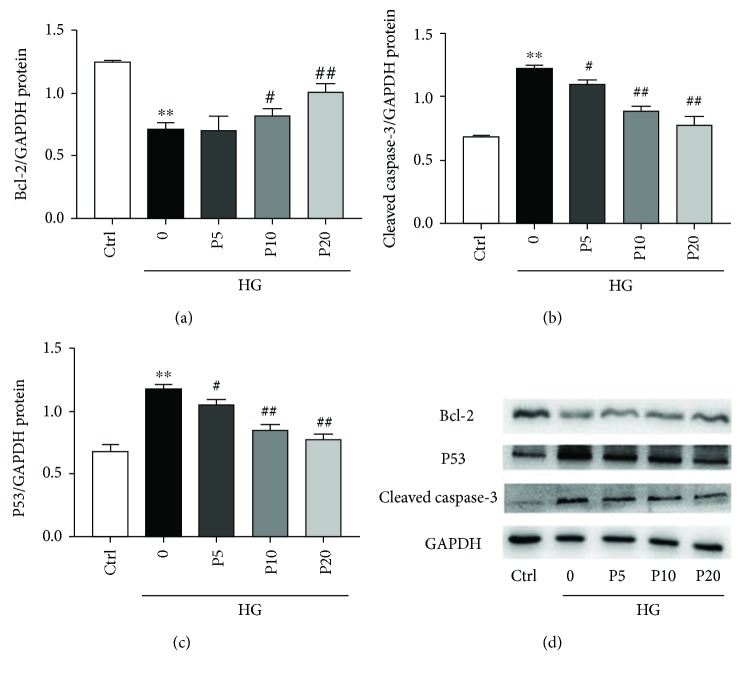
The effects of propofol on the expression of apoptosis-related proteins in H9c2 cells during high glucose stress. The levels of Bcl-2, cleaved caspase-3, and P53 were determined by western blotting, and GAPDH was used as positive control. (a) The effects of propofol on Bcl-2 expression. (b) The effects of propofol on cleaved caspase-3 expression. (c) The effects of propofol on P53 expression. Data were shown as mean ± SEM from five independent experiments. ^∗^*P* < 0.05, ^∗∗^*P* < 0.01, and ^∗∗∗^*P* < 0.001 versus control; ^#^*P* < 0.05, ^##^*P* < 0.01, and ^###^*P* < 0.001 versus the high glucose-treated group without drugs. Ctrl = blank control group; 0 = high glucose model group; P5 = 5 *μ*M propofol pretreatment group; P10 = 10 *μ*M propofol pretreatment group; P20 = 20 *μ*M propofol pretreatment group; and HG = 22 mM high glucose culture.

Annexin V-PE is a sensitive probe for identifying apoptotic cells. Annexin V-positive staining cells (Q2 and Q3) have been regarded as undergoing apoptosis. Our data showed that high glucose treatment induces apoptosis in the H9c2 cells, whereas treatment of propofol (5, 10, and 20 *μ*M) for 72 h markedly decreases the apoptosis rates dose-dependently (Figure 3). We also found that the level of Bcl-2, an antiapoptotic protein, is inhibited by high glucose stress and propofol increases it dose-dependently (Figures 4(a) and 4(d)). In consistent, the levels of cleaved caspase-3 and p53, markers of apoptosis, were increased with high glucose and propofol decreased them dose-dependently (Figures 4(b)–4(d)). These results demonstrated that propofol can inhibit cellular apoptosis induced by high glucose stress.

### 3.6. Propofol Alleviated Apoptosis Induced by High Glucose Stress through the Regulation of SIRT3, SOD2, PHD2, and HIF-1*α* in H9c2 Cells

**Figure 5:**
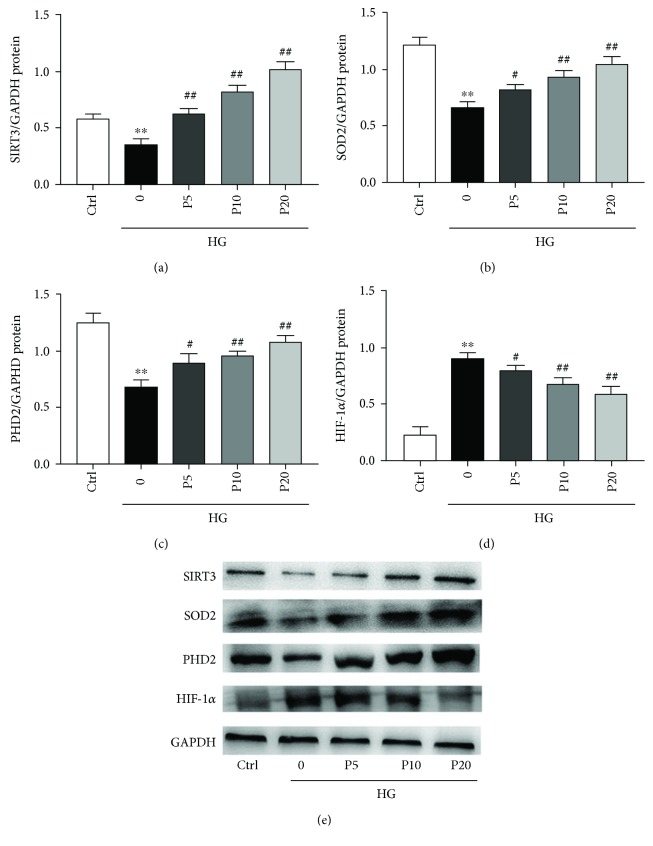
The effects of propofol on the levels of SIRT3, SOD2, PHD2, and HIF-1*α* in H9c2 cells during high glucose stress. The levels of SIRT3, SOD2, PHD2, and HIF-1*α* were determined by western blotting, and GAPDH was used as positive control. The quantification of the effects of propofol on protein levels of SIRT3 (a), SOD2 (b), PHD (c), and HIF-1*α* (d). Data were shown as mean ± SEM from five independent experiments. ^∗^*P* < 0.05, ^∗∗^*P* < 0.01, and ^∗∗∗^*P* < 0.001 versus control; ^#^*P* < 0.05, ^##^*P* < 0.01, and ^###^*P* < 0.001 versus the high glucose-treated group without drugs. Ctrl = blank control group; 0 = high glucose model group; P5 = 5 *μ*M propofol pretreatment group; P10 = 10 *μ*M propofol pretreatment group; P20 = 20 *μ*M propofol pretreatment group; and HG = 22 mM high glucose culture.

We evaluated target protein expression by using the relative grey value/GAPDH in each group. Compared to the control group, high glucose significantly decreased the protein levels of SIRT3, SOD2, and PHD2 but elevated the protein level of HIF-1*α*. As expected, compared to the model group, the levels of SIRT3, SOD2, and PHD2 in the propofol groups were increased and the level of HIF-1*α* was decreased in a dose-dependent manner (Figures 5(a)–5(e)).

### 3.7. Effects of Propofol on the mRNA of HIF-1*α* and PHD2 in H9c2 Cells during High Glucose Stress

**Figure 6:**
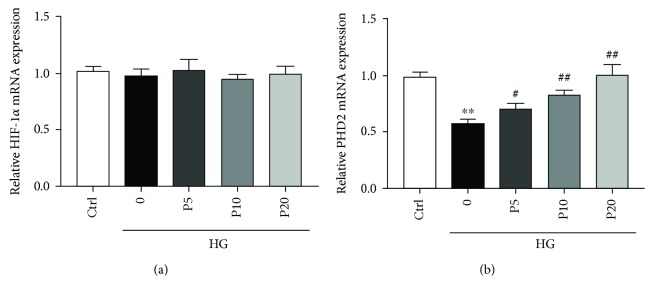
The effects of propofol on the mRNA levels of HIF-1*α* and PHD2 in H9c2 cells during high glucose stress. (a) The relative mRNA expressions of HIF-1*α*. (b) The relative mRNA expressions of PHD2. Data were shown as mean ± SEM from five independent experiments. ^∗^*P* < 0.05, ^∗∗^*P* < 0.01, and ^∗∗∗^*P* < 0.001 versus control; ^#^*P* < 0.05, ^##^*P* < 0.01, and ^###^*P* < 0.001 versus the high glucose-treated group without drugs. Ctrl = blank control group; 0 = high glucose model group; P5 = 5 *μ*M propofol pretreatment group; P10 = 10 *μ*M propofol pretreatment group; P20 = 20 *μ*M propofol pretreatment group; P40 = 40 *μ*M propofol pretreatment group; and HG = 22 mM high glucose culture.

To further characterize the mechanisms leading to reduced level of HIF-1*α* during propofol treatment, we first analyzed the gene expression of HIF-1*α*. Q-PCR analysis showed that treatment with or without propofol did not significantly change HIF-1*α* mRNA levels in H9c2 cardiomyoblasts during high glucose stress, when compared to the control group (Figure 6(a)). This raised the question whether decreased level of HIF-1*α* protein is a result of increased protein degradation, given the fact that propofol increased protein level of PHD2, a HIF prolyl hydroxylase that regulates the degradation of HIF protein by proteolysis. So, we analyzed the mRNA level of PHD2. Our results showed that in consistent with the protein levels, high glucose significantly decreases PHD2 mRNA level in H9c2 cells, whereas treatment of propofol increased PHD2 mRNA levels dose-dependently compared to the high glucose group (Figure 6(b)). These results showed that propofol treatment seems to decrease HIF-1*α* protein levels by increasing proline hydroxylase-dependent degradation rather than by inhibiting transcription during high glucose stress.

### 3.8. The Inhibition of HIF-1*α* Expression Decreased Apoptosis and Inflammation in H9c2 Cells during High Glucose Stress

**Figure 7:**
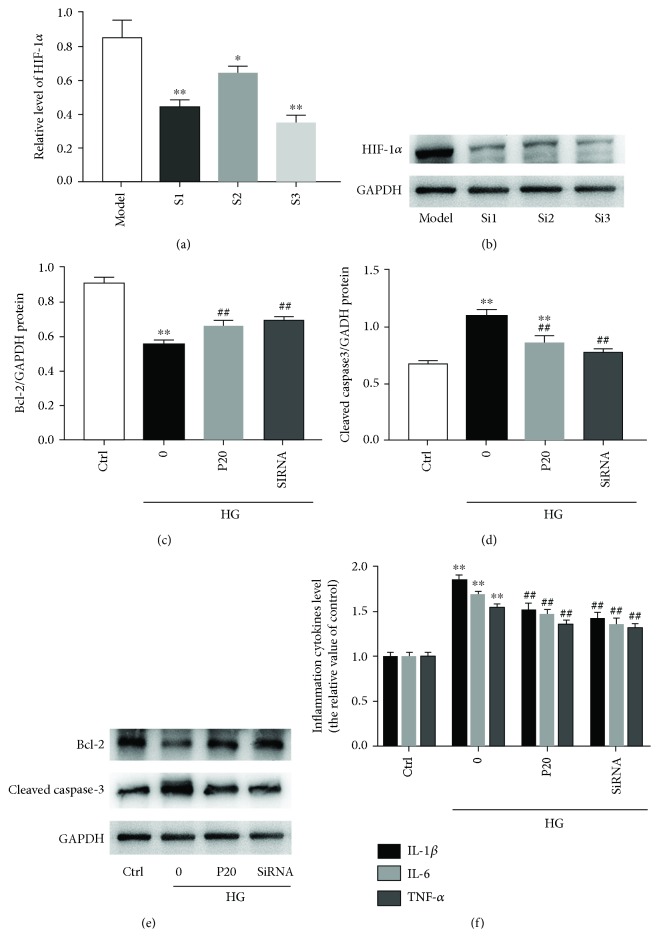
The HIF-1*α* silence mimicked the effect of propofol in H9c2 cells during high glucose stress. (a, b) Representative image of HIF-1*α* silence vesicles in H9c2 cells, which was, respectively, transfected with 50 nM siRNA-HIF-1*α*-1, siRNA-HIF-1*α*-2, or siRNA-HIF-1*α*-3. (c, d) Levels of Bcl-2 and cleaved caspase-3 in the high glucose-induced and siRNA-transfected H9c2 cells. (e) Western blotting of Bcl-2 and cleaved caspase-3 in H9c2 cells treated with 20 *μ*M propofol or HIF-1*α* silence. (f) Levels of proinflammatory cytokines in H9c2 cells treated with 20 *μ*M propofol or HIF-1*α* silence. Data were shown as mean ± SEM from five independent experiments. ^∗^*P* < 0.05, ^∗∗^*P* < 0.01, and ^∗∗∗^*P* < 0.001 versus control; ^#^*P* < 0.05, ^##^*P* < 0.01, and ^###^*P* < 0.001 versus the high glucose-treated group without drugs. Model = high glucose model group; S1 = siRNA-HIF-1*α*-1 group; S2 = SiRNA-HIF-1*α*-2 group; S3 = SiRNA-HIF-1*α*-3 group; Ctrl = blank control group; 0 = high glucose model group; P20 = 20 *μ*M propofol pretreatment group; SiRNA = HIF-1*α* silence group; and HG = 22 mM high glucose culture.

To further elucidate the role of HIF-1*α* in the high glucose stress damage in H9c2 cells, we silenced HIF-1*α* by using HIF-1*α*-specific siRNAs and then reevaluated the effects of propofol on high glucose-induced apoptosis and inflammation responses. Three pairs of siRNA-HIF-1*α* were transfected onto the cells and we found that transfection with 50 nM siRNA-HIF-1*α*-3 markedly downregulates both the mRNA and protein levels of HIF-1*α* (Figures 7(a) and 7(b)). Interestingly, after the significant silence of HIF-1*α* gene, similar changes in the levels of apoptosis regulatory proteins were observed (Figures 7(c)–7(e)). And the levels of proinflammatory cytokines in the media in the siRNA-HIF-1*α*-transfected H9c2 cells were also decreased during high glucose stress (Figure 7(f)).

## 4. Discussion

Both *in vivo* and *in vitro* studies have shown that propofol had protective effects against high glucose injury [13, 18, 19]. However, the mechanism of the cardioprotective effect of propofol under high glucose stress remains poorly elucidated. Here, our data revealed that propofol decreases cell apoptosis induced by high glucose stress through the inhibition of ROS and production of proinflammatory cytokines. Our data also showed that these effects are at least partially mediated through the induction of HIF-1*α* degradation via upregulation of the SIRT3 and PHD2 signal pathways.

Increased oxidative stress is found to be associated with type 2 diabetes and its complications. To investigate the mechanism of the protective effect of propofol on myocardial hyperglycemic injury, an *in vitro* model of myocardial cell injury induced by high glucose was established. We showed that treatment of propofol rescues the high glucose-induced reduction in cell viability. The increased cell viability could be a result from decreased cell death and/or induction of proliferation. We first looked into the apoptosis rate in propofol-treated cells, as it is known that high glucose induces apoptosis. We demonstrated that treatment with propofol (5-20 *μ*M) reduces cardiomyoblast apoptosis, and this is associated with attenuated levels of ROS via increase of ROS scavenging during high glucose stress in a dose-dependent manner. Compared with MTT assays, flow cytometry which can distinguish between apoptotic and necrotic cells is a classical method for detecting cell apoptosis with high sensitivity [20]. We also demonstrated that propofol inhibited cell apoptosis during high glucose injury, which is consistent with the previous study showing that propofol has protective effect on cardiac myocytes [13].

**Figure 8:**
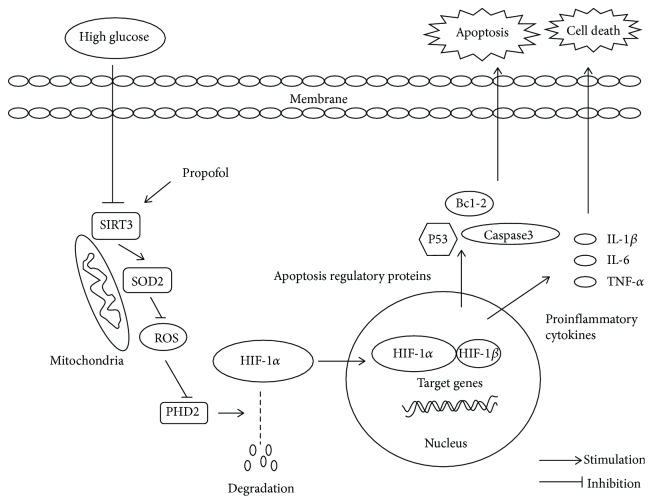
Diagram of possible protective effect of propofol via regulation of HIF-1*α* during high glucose stress.

As shown in Figure 8, one of the most important insults from inflammation and oxidative stress induced by high glucose stress is apoptosis of H9c2 cells [21]. The importance of inflammation in pathogenesis of diabetic cardiomyopathy is well recognized. A growing body of evidence has demonstrated that inflammation response also occurs in the myocardium of diabetic patients [22]. The role of proinflammatory cytokines such as IL-6, IL-1*β*, and TNF-*α* in acute high glucose-induced cardiotoxicity has also been shown in an *in vitro* model [23]. Previous studies have shown that propofol inhibits inflammatory factor release in mononuclear cells [24] and in microglia cells [25]. These findings lead us to speculate that propofol might also protect the cells against high glucose-induced inflammation. Indeed, our results showed that high-glucose stimulation successfully induces inflammation in H9c2 cells as evidenced by the increased level of IL-6, IL-1*β*, and TNF-*α* in the media. Interestingly, propofol attenuated the high glucose-induced inflammation response by reducing the production of proinflammatory cytokines.

To further explore the mechanisms by which propofol exerts antiapoptotic effects, we studied the markers and inducers of cellular apoptosis. Bcl-2 is an antiapoptotic protein; when it is highly expressed, the apoptosis of cells is mostly inhibited [26]. Our study showed that high glucose induced apoptosis by downregulating the protein level of antiapoptotic Bcl-2 and upregulating proapoptotic proteins, such as cleaved caspase-3. In contrast, cells that pretreated with propofol showed upregulated levels of Bcl-2 and downregulated levels of cleaved caspase-3 compared to the high glucose group, indicating that propofol protects cells from apoptosis by modulating anti- and proapoptotic proteins. p53 is involved in cell cycle arrest, cellular senescence, and apoptosis via inhibiting proliferation. It has been shown that hyperglycemia-induced cardiomyocyte apoptosis is associated with the elevation of p53 [27]. In consistent with these findings, we also demonstrated that high glucose increases protein level of p53 in H9c2 cells, whereas propofol decreases the level of p53 induced by high glucose exposure. This indicates that the suppression of p53 by propofol should also be taken into account in the role of propofol in the inhibition of high glucose-induced cardiomyocyte apoptosis.

Sirtuins are NAD-dependent deacetylases that share homology to the yeast Sir2 protein. Of the three mitochondrial sirtuins, SIRT3 is the most well studied to date and has been long considered as a tumor suppressor by activating manganese superoxide dismutase (MnSOD), a mitochondrial antioxidant enzyme, decreasing ROS levels and maintaining the stabilization of HIF-1*α* [28, 29]. Recent studies highlight that expression and activity of SIRT3 are decreased in type 2 diabetes, which is associated with defects in glucose tolerance [30]. In contrast, overexpression of SIRT3 antagonizes high glucose-induced apoptosis in renal tubular epithelial cells [31]. Based on these, we postulated that the protective role of propofol in high glucose-induced mitochondrial oxidative stress and apoptosis is mediated through the activation of SIRT3. Indeed, in the present study, we found that there was a significant decrease in protein level of SIRT3 and SOD2 in H9c2 cells after high glucose exposure, and propofol reversed it. We also observed the high protein level of HIF-1*α* in H9c2 cells during high glucose stress. Two independent studies have demonstrated that SIRT3 destabilizes HIF-1*α* by promoting prolyl hydroxylase (PHD) via a mechanism dependent on a decrease in cellular ROS levels under normoxia [29, 32]. Therefore, we speculate that propofol may alleviate high glucose-induced apoptosis through SIRT3-mediated HIF-1*α* destabilization in H9c2 cells (Figure 7). Since previous studies indicated that HIF-1*α* plays a central role in regulating the glucose utilization in myocardial cells [33], we think that SIRT3 may create a cellular environment favoring the development of genomic stability by regulating HIF-1*α* protein level during high glucose stress.

HIF-1*α* is a key transcription factor that controls the adaptive response to hypoxic conditions in the cell through regulating downstream target genes [34]. Normally, activity of HIF-1*α* is depending on the amount of HIF-1*α* protein, which is markedly induced under hypoxia condition, whereas HIF-1*β* protein is constitutively expressed in the cells regardless of oxygen tension [35]. The protein level of HIF-1*α* is determined by the kinetic balance between protein synthesis and degradation. Under normoxia condition, the proline residue in the oxygen-dependent degradation (ODD) domain of HIF-1*α* is hydroxylated by PHD, which promotes the interaction of HIF-1*α* and von Hippel-Lindau protein-elongin B-elongin C complex, leading to the ubiquitination and degradation in the proteasome with a very short half-life [36, 37]. On the contrary, hypoxia impairs PHD activity and suppresses the hydroxylation of HIF-1*α*, resulting in reduced HIF-1*α* degradation and increased accumulation of HIF-1*α*, which subsequently increases of target gene transcription. It has been shown that in addition to the hypoxic stimuli, many other factors regulate HIF-1*α* and PHD expression even under normoxic condition, for example, metabolic disturbance and oxidative stress [38–41]. HIF-1*α* is usually regulated not only at the protein level but also at the transcription level [42]. To investigate the mechanism that causes decreased protein level of HIF-1*α* accumulation during propofol treatment, we analyzed both mRNA and protein levels of HIF-1*α* in H9c2 cells during high glucose insult. We found that propofol has no significant effect on HIF-1*α* mRNA but significantly reduces the protein level of HIF-1*α*, suggesting the regulation of propofol on HIF-1*α* happened on the posttranslational but not on the transcriptional level. In addition, our data also showed that compared to the control group, high glucose induces significantly decrease in both mRNA and protein levels of PHD2, whereas treatment of propofol greatly increases PHD2 levels in a dose-dependent manner compared to the high glucose only group. This, together with the reduced protein but not mRNA of HIF-1*α*, indicates that propofol enhances the degradation process of HIF-1*α* through the increase in both mRNA and protein levels of PHD2 in H9c2 cells.

HIF-1*α* controls over 100 target genes involving angiogenesis, metabolic adaptation, inflammation, and apoptosis through directly binding to the promoter region of the targets. HIF-1*α* stabilization is critical to LPS-induced IL-1*β* expression in macrophages [43]. HIF-1*α* deletion in myeloid cells led to reduced proinflammatory cytokines such as IL-1, IL-12, and TNF-*α* in the rat model of sepsis [44]. Furthermore, HIF-1*α* can induce apoptosis via increasing the stability of p53 under hypoxia condition [45]. The increase of HIF-1*α* can also downregulate the expression of Bcl-2, an antiapoptotic protein, which in turn induces apoptosis of human umbilical vein endothelial cells during hypoxia [46], whereas the inhibition of HIF-1*α* expression can reduce cleaved caspase-3 expression in the rat model of hemorrhagic shock and alleviate the acute lung injury in rats [47]. Given that persistent expression of HIF-1*α* is an important factor for the deterioration of cell viability during high glucose exposure, we detected the changes of antiapoptotic and proapoptotic proteins including Bcl-2 and cleaved caspase-3 after knockdown of HIF-1*α* suing siRNA. We showed that, compared to high glucose only group, knockdown of HIF-1*α* significantly decreases the level of cleaved caspase-3 and increases protein level of Bcl-2, suggesting reduction of HIF-1*α* diminishes apoptosis induced by high glucose stress. Interestingly, we found a comparable effect of propofol on these proteins in H9c2 cells after high glucose exposure, indicating that the effects of propofol may be mediated through HIF-1*α*. Our results also indicate that high glucose-induced apoptosis in cardiomyocytes is mediated through the high elevated expression of HIF-1*α*. We and others have shown that increased proinflammatory cytokine secretion is associated with the elevated apoptosis induced by high glucose. To further define whether the regulation of HIF-1*α* by propofol plays a vital role on cytokine release during high glucose exposure, we examined the proinflammatory cytokines by ELISA after knocking down of HIF-1*α*. We observed that HIF-1*α* silence plays a similar protective effect with propofol on suppressing proinflammatory cytokine secretion after high glucose exposure. Since we think HIF-1*α* is the major transcriptional factor that regulates high glucose-induced apoptosis and cytokine secretion, it is not surprising to see that HIF-1*α* knockdown has more significant effect on IL-6, TNF-*α*, and IL-1*β* secretion than propofol treatment.

To further explore the effect of HIF-1*α* on cell damage induced by high glucose stress in H9c2 cell, we evaluated the inhibitor of PHD on cell viability before or after the high glucose stress. Since the transfection time of HIF-1*α* siRNA needs 6 hours, we also chose 6 hours as preincubation or after incubation time to evaluate the effect of IOX2 during high glucose stress. Preincubation with IOX2 significantly decrease the cell damage during high glucose stress, while IOX2 significantly aggravated cell damage after 6 hours of retardation time during high glucose insult. These results suggest that expression of HIF-1*α* at the given window stage of oxidative stress may play a pivotal role on cell survival during oxidative stress.

Despite the interesting findings, our study has several limitations. Firstly, we just use mice H9c2 cells which are nonhuman cells in our experiments. It should be prudent to extrapolate it to human entity. Secondly, we only focused on HIF-1 but not HIF-2 due to previous description that HIF-1 regulates glucose metabolism in the control of apoptosis signaling and HIF-2 functions as an important regulator of hepatic lipid metabolism.

## 5. Conclusion

Collectively, our current study demonstrated that propofol protects cardiomyocytes from high glucose-induced apoptosis and proinflammatory cytokine production through the regulation of HIF-1*α* protein stability. The increased HIF-1*α* degradation and decreased protein level are due to the upregulation of SIRT3 and ROS scavenging process, which in turn induces the mRNA and protein levels of a HIF prolyl hydroxylase, PHD2 in H9c2 cells. IOX2 significantly inhibited cell viability during high glucose stress. Our study highlights the potential therapeutic effect of propofol against high glucose-induced injury in rodent cardiomyocytes by targeting on the ROS scavenging and HIF-1*α* signaling pathways.

## Figures and Tables

**Table 1:** Primer sequences for real-time PCR.

	Forward primer	Reverse primer
*HIF-1α*	ACACAGAAATGGCCCAGTGAG	CACCTTCCACGTTGCTGACTT
*PHD2*	GCCAAGGTGAGCGGAGGTATT	TTGCGTACCTTGTGGCGTATG
*GAPDH*	TTCCTACCCCCAATGTATCCG	CATGAGGTCCACCACCCTGTT

**Table 2:** The sequence of siRNAs.

	Sense strand	Antisense strand
*Rn-HIF-1α-si-1*	5′-GGAGGACGAUGAACAUCAAdTdT-3′	5′-UUGAUGUUCAUCGUCCUCCdTdT-3′
*Rn-HIF-1α-si-2*	5′-GCAUUGAAGUUAGAGUCAAdTdT-3′	5′-UUGACUCUAACUUCAAUGCdTdT-3′
*Rn-HIF-1α-si-3*	5-′GUGUGUGAAUUAUGUUGUATdT-3′	5′-UACAACAUAAUUCACACACdTdT-3′

## Data Availability

The experiment data used to support the findings of this study are included within the article.

## References

[1] Cho N. H., Shaw J. E., Karuranga S. (2018). IDF Diabetes Atlas: global estimates of diabetes prevalence for 2017 and projections for 2045. *Diabetes Research and Clinical Practice*.

[2] Ford E. S. (2005). Risks for all-cause mortality, cardiovascular disease, and diabetes associated with the metabolic syndrome: a summary of the evidence. *Diabetes Care*.

[3] O'Keefe J. H., Bell D. S. H. (2007). Postprandial hyperglycemia/hyperlipidemia (postprandial dysmetabolism) is a cardiovascular risk factor. *The American Journal of Cardiology*.

[4] Eriksson U. J., Borg L. A. H. (1993). Diabetes and embryonic malformations. Role of substrate-induced free-oxygen radical production for dysmorphogenesis in cultured rat embryos. *Diabetes*.

[5] Nishikawa T., Edelstein D., du X. L. (2000). Normalizing mitochondrial superoxide production blocks three pathways of hyperglycaemic damage. *Nature*.

[6] Falcao-Pires I., Leite-Moreira A. F. (2012). Diabetic cardiomyopathy: understanding the molecular and cellular basis to progress in diagnosis and treatment. *Heart Failure Reviews*.

[7] Drimal J., Knezl V., Navarova J. (2008). Role of inflammatory cytokines and chemoattractants in the rat model of streptozotocin-induced diabetic heart failure. *Endocrine Regulations*.

[8] Rajamani U., Essop M. F. (2010). Hyperglycemia-mediated activation of the hexosamine biosynthetic pathway results in myocardial apoptosis. *American Journal of Physiology-Cell Physiology*.

[9] Vasileiou I., Xanthos T., Koudouna E. (2009). Propofol: a review of its non-anaesthetic effects. *European Journal of Pharmacology*.

[10] Liu Q., Yao J. Y., Qian C. (2012). Effects of propofol on ischemia-induced ventricular arrhythmias and mitochondrial ATP-sensitive potassium channels. *Acta Pharmacologica Sinica*.

[11] Li W., Zhang Y., Liu Y. (2012). In vitro kinetic evaluation of the free radical scavenging ability of propofol. *Anesthesiology*.

[12] Chen R. M., Chen T. G., Chen T. L. (2005). Anti-inflammatory and antioxidative effects of propofol on lipopolysaccharide-activated macrophages. *Annals of the New York Academy of Sciences*.

[13] Xu J., Li H., Irwin M. G. (2014). Propofol ameliorates hyperglycemia-induced cardiac hypertrophy and dysfunction via heme oxygenase-1/signal transducer and activator of transcription 3 signaling pathway in rats. *Critical Care Medicine*.

[14] Finkel T., Deng C. X., Mostoslavsky R. (2009). Recent progress in the biology and physiology of sirtuins. *Nature*.

[15] Yang F., Zhou L., Wang D., Wang Z., Huang Q. Y. (2015). Minocycline ameliorates hypoxia-induced blood-brain barrier damage by inhibition of HIF-1*α* through SIRT-3/PHD-2 degradation pathway. *Neuroscience*.

[16] Liu G., Cao M., Xu Y., Li Y. (2015). SIRT3 protects endothelial cells from high glucose-induced cytotoxicity. *International Journal of Clinical and Experimental Pathology*.

[17] Zhao D., Li Q., Huang Q. (2015). Cardioprotective effect of propofol against oxygen glucose deprivation and reperfusion injury in H9c2 cells. *Oxidative Medicine and Cellular Longevity*.

[18] Zhu M., Wen M., Sun X., Chen W., Chen J., Miao C. (2015). Propofol protects against high glucose-induced endothelial apoptosis and dysfunction in human umbilical vein endothelial cells. *Anesthesia and Analgesia*.

[19] Zhu M., Chen J., Tan Z., Wang J. (2012). Propofol protects against high glucose-induced endothelial dysfunction in human umbilical vein endothelial cells. *Anesthesia and Analgesia*.

[20] Meyer M., Essack M., Kanyanda S., Rees J. G. (2008). A low-cost flow cytometric assay for the detection and quantification of apoptosis using an anionic halogenated fluorescein dye. *BioTechniques*.

[21] Das J., Ghosh J., Manna P., Sinha M., Sil P. C. (2009). Taurine protects rat testes against NaAsO_2_-induced oxidative stress and apoptosis via mitochondrial dependent and independent pathways. *Toxicology Letters*.

[22] Madonna R., Geng Y. J., Bolli R. (2014). Co-activation of nuclear factor-*κ*B and myocardin/serum response factor conveys the hypertrophy signal of high insulin levels in cardiac myoblasts. *Journal of Biological Chemistry*.

[23] Xu W., Chen J., Lin J. (2015). Exogenous H2S protects H9c2 cardiac cells against high glucose-induced injury and inflammation by inhibiting the activation of the NF-*κ*B and IL-1*β* pathways. *International Journal of Molecular Medicine*.

[24] Tang J., Chen X., Tu W. (2011). Propofol inhibits the activation of p38 through up-regulating the expression of annexin A1 to exert its anti-inflammation effect. *PLoS One*.

[25] Lu Y., Gu Y., Ding X., Wang J., Chen J., Miao C. (2017). Intracellular Ca^2+^ homeostasis and JAK1/STAT3 pathway are involved in the protective effect of propofol on BV2 microglia against hypoxia-induced inflammation and apoptosis. *PLoS One*.

[26] Villar J., Quadri H. S., Song I., Tomita Y., Tirado O. M., Notario V. (2009). PCPH/ENTPD5 expression confers to prostate cancer cells resistance against cisplatin-induced apoptosis through protein kinase C*α*–mediated Bcl-2 stabilization. *Cancer Research*.

[27] Fiordaliso F., Leri A., Cesselli D. (2001). Hyperglycemia activates p53 and p53-regulated genes leading to myocyte cell death. *Diabetes*.

[28] Chen Y., Zhang J., Lin Y. (2011). Tumour suppressor SIRT3 deacetylates and activates manganese superoxide dismutase to scavenge ROS. *EMBO Reports*.

[29] Finley L. W. S., Carracedo A., Lee J. (2011). SIRT3 opposes reprogramming of cancer cell metabolism through HIF1*α* destabilization. *Cancer Cell*.

[30] Dong X. C. (2012). Sirtuin biology and relevance to diabetes treatment. *Diabetes Management*.

[31] Jiao X., Li Y., Zhang T., Liu M., Chi Y. (2016). Role of sirtuin3 in high glucose-induced apoptosis in renal tubular epithelial cells. *Biochemical and Biophysical Research Communications*.

[32] Bell E. L., Emerling B. M., Ricoult S. J. H., Guarente L. (2011). SirT3 suppresses hypoxia inducible factor 1*α* and tumor growth by inhibiting mitochondrial ROS production. *Oncogene*.

[33] Wang F., Liang G. Y., Liu D. X. (2015). Effect of Si-RNA-silenced HIF-1*α* gene on myocardial ischemia-reperfusion-induced insulin resistance. *International Journal of Clinical and Experimental Medicine*.

[34] Hirota K. (2002). Hypoxia-inducible factor 1, a master transcription factor of cellular hypoxic gene expression. *Journal of Anesthesia*.

[35] Huang L. E., Arany Z., Livingston D. M., Bunn H. F. (1996). Activation of hypoxia-inducible transcription factor depends primarily upon redox-sensitive stabilization of its *α* subunit. *Journal of Biological Chemistry*.

[36] Salceda S., Caro J. (1997). Hypoxia-inducible factor 1*α* (HIF-1*α*) protein is rapidly degraded by the ubiquitin-proteasome system under normoxic conditions. Its stabilization by hypoxia depends on redox-induced changes. *Journal of Biological Chemistry*.

[37] Masoud G. N., Li W. (2015). HIF-1*α* pathway: role, regulation and intervention for cancer therapy. *Acta Pharmaceutica Sinica B*.

[38] Lu X., Kang Y. (2010). Hypoxia and hypoxia-inducible factors: master regulators of metastasis. *Clinical Cancer Research*.

[39] Treins C., Giorgetti-Peraldi S., Murdaca J., van Obberghen E. (2001). Regulation of vascular endothelial growth factor expression by advanced glycation end products. *Journal of Biological Chemistry*.

[40] Gao Q., Guan L., Hu S. (2015). Study on the mechanism of HIF1a-SOX9 in glucose-induced cardiomyocyte hypertrophy. *Biomedicine & Pharmacotherapy*.

[41] Feldser D., Agani F., Iyer N. V., Pak B., Ferreira G., Semenza G. L. (1999). Reciprocal positive regulation of hypoxia-inducible factor 1*α* and insulin-like growth factor 2. *Cancer Research*.

[42] Townley-Tilson W. H. D., Pi X., Xie L. (2015). The role of oxygen sensors, hydroxylases, and HIF in cardiac function and disease. *Oxidative Medicine and Cellular Longevity*.

[43] Tannahill G. M., Curtis A. M., Adamik J. (2013). Succinate is an inflammatory signal that induces IL-1*β* through HIF-1*α*. *Nature*.

[44] Peyssonnaux C., Cejudo-Martin P., Doedens A., Zinkernagel A. S., Johnson R. S., Nizet V. (2007). Cutting edge: essential role of hypoxia inducible factor-1*α* in development of lipopolysaccharide-induced sepsis. *Journal of Immunology*.

[45] Chen D., Li M., Luo J., Gu W. (2003). Direct interactions between HIF-1*α* and Mdm2 modulate p53 function. *Journal of Biological Chemistry*.

[46] Iida T., Mine S., Fujimoto H., Suzuki K., Minami Y., Tanaka Y. (2002). Hypoxia-inducible factor-1*α* induces cell cycle arrest of endothelial cells. *Genes to Cells*.

[47] Jiang H., Huang Y., Xu H., Hu R., Li Q. F. (2012). Inhibition of hypoxia inducible factor-1*α* ameliorates lung injury induced by trauma and hemorrhagic shock in rats. *Acta Pharmacologica Sinica*.

